# Antiemetics for reducing vomiting related to acute gastroenteritis in children and adolescents

**DOI:** 10.1590/S1516-31802012000400015

**Published:** 2012-09-04

**Authors:** Zbys Fedorowicz, Vanitha A. Jagannath, Ben Carter

## Abstract

**BACKGROUND:**

Vomiting is a common manifestation of acute gastroenteritis in children and adolescents. When untreated, it can be a hindrance to oral rehydration therapy, which is the cornerstone in the management of acute gastroenteritis. Evidence is needed concerning the safety and efficacy of antiemetic use for vomiting in acute gastroenteritis in children.

**OBJECTIVES:**

To assess the safety and effectiveness of antiemetics on gastroenteritis induced vomiting in children and adolescents.

**SEARCH STRATEGY:**

We searched the Cochrane Upper Gastrointestinal and Pancreatic Diseases Group Trials Register comprising references identified from comprehensive electronic database searches and hand searches of relevant journals and abstract books of conferences. The search was re-run and is up to date as on 20 July 2010.

**SELECTION CRITERIA:**

Randomized controlled trials comparing antiemetics with placebo or no treatment, in children and adolescents under the age of 18, for vomiting due to gastroenteritis.

**DATA COLLECTION AND ANALYSIS:**

Two review authors independently assessed trial quality and extracted data.

**MAIN RESULTS:**

We included seven trials involving 1,020 participants. Mean time to cessation of vomiting in one study was 0.34 days less with dimenhydrinate suppository compared to placebo (P value = 0.036). Pooled data from three studies comparing oral ondansetron with placebo showed: a reduction in the immediate hospital admission rate (RR 0.40, NNT 17, 95% CI 10 to 100) but no difference between the hospitalization rates at 72 hours after discharge from the Emergency Department (ED); a reduction in IV rehydration rates both during the ED stay (RR 0.41, NNT 5, 95% CI 4 to 8), and in follow-up to 72 hours after discharge from the ED stay (worst-best scenario for ondansetron RR 0.57, NNT 6, 95% CI 4 to 13) and an increase in the proportion of patients with cessation of vomiting (RR 1.34, NNT 5, 95% CI 3 to 7). No significant difference was noted in the revisit rates or adverse events, although diarrhea was reported as a side effect in four of the five ondansetron studies. In one study the proportion of patients with cessation of vomiting in 24 hours was (58%) with IV ondansetron, (17%) placebo and (33%) in the metoclopramide group (P value = 0.039).

**AUTHORS’ CONCLUSIONS:**

Oral ondansetron increased the proportion of patients who had ceased vomiting and reduced the number needing intravenous rehydration and immediate hospital admission. Intravenous ondansetron and metoclopramide reduced the number of episodes of vomiting and hospital admission, and dimenhydrinate as a suppository reduced the duration of vomiting.

**This is the abstract of a Cochrane Review published in the Cochrane Database of Systematic Reviews (CDSR) 2011, Issue 9, Art. No.: CD005506. DOI:** 10.1002/14651858.CD005506.pub5 (http://onlinelibrary.wiley.com/doi/10.1002/14651858.CD005506.pub5/abstract). For full citation and authors’ details see reference 1.

**The full text is freely available from:** http://www.cochranejournalclub.com/antiemetics-reducing-vomiting-related-acute-gastroenteritis-c/pdf/CD005506.pdf
